# Primary Acinic Cell Carcinoma of the Breast: A Clinicopathological and Immunohistochemical Study

**DOI:** 10.1155/2013/372947

**Published:** 2013-09-26

**Authors:** Kiyoshi Shingu, Tokiko Ito, Gengo Kaneko, Nobuo Itoh

**Affiliations:** Departments of Surgery and Pathology, Iida Municipal Hospital, 438 Yawatacho, Iida, Nagano 395-8502, Japan

## Abstract

Acinic cell carcinoma of the breast is an extremely rare, malignant neoplasm characterized by widespread acinar cell-like differentiation and clinically low-grade malignancy. Herein, we report a case of acinic cell carcinoma of the breast in a 41-year-old woman. The tumor was poorly demarcated but had a firm consistency. It was removed with lumpectomy, and sentinel lymph node biopsy was performed to check for metastasis. Microscopically, the tumor showed an infiltrative growth pattern with a combination of solid, trabecular, and microglandular areas. Many of the tumor cells had abundant clear vacuolated cytoplasm containing zymogen-typed granules which resemble acinar cells of the salivary glands. The immunohistochemical profile of the tumor was also similar to that of salivary gland acinic cell carcinoma: the tumor cells were positive for amylase, lysozyme, **α**-1-antichymotrypsin, S-100 protein, and epithelial membrane antigen and negative for estrogen receptor, progesterone receptor, and human epidermal growth factor receptor 2. She received postoperative chemoradiation therapy and has been well for 3 years since surgery. As studies on large series are lacking, further studies are needed to elucidate the biological characteristics of acinic cell carcinoma of the breast.

## 1. Introduction

Acinic cell carcinomas (ACCs) of the breast are similar to tumors that occur in the salivary glands and show acinic cell differentiation. This tumor type was first described in 1996 by Roncaroli et al. [1], and since then, several such cases have been reported [2–10]. Although it is an extremely rare tumor, its histological, immunohistochemical, and, ultrastructural characteristics have been described in previous reports [2–10]. Herein, we report a case of pure acinic cell carcinoma of the breast in a 41-year-old Japanese woman and review the English literature we could obtain.

## 2. Case Report

**Figure 1:**
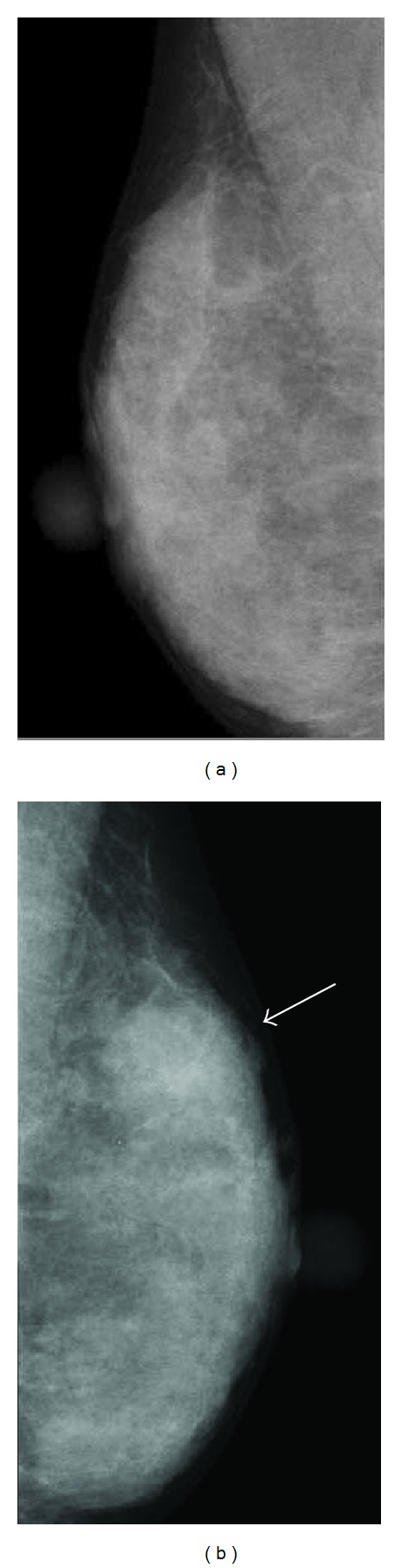
Mammography revealed a focal asymmetric density (arrow) in the lateral upper quadrant of the left breast.

**Figure 2:**
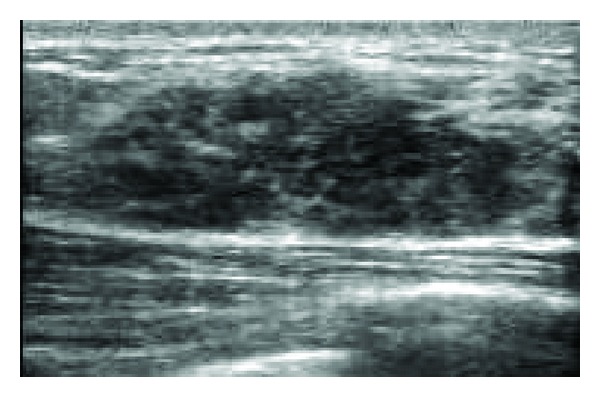
Ultrasonography showed a heterogeneous hypoechoic mass with an ill-defined margin, measuring 3.4 × 1.1 × 3.0 cm.

**Figure 3:**
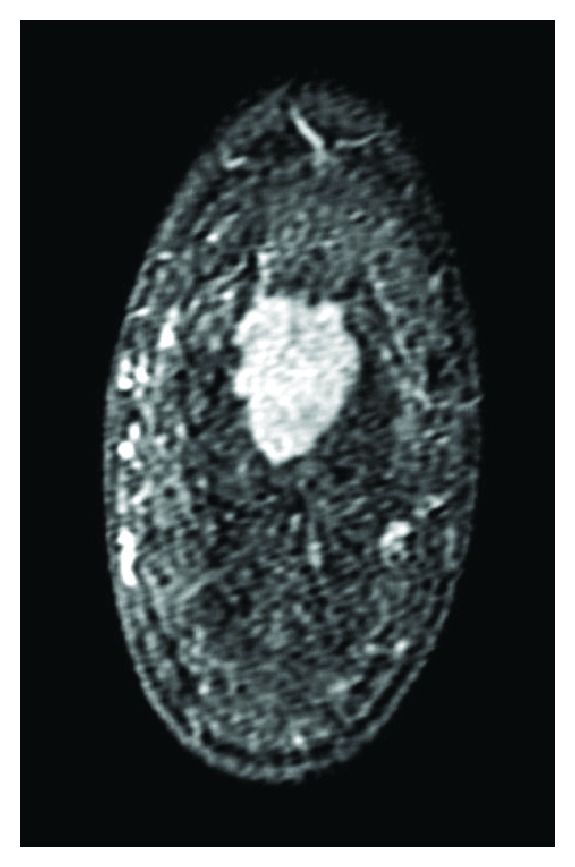
Enhanced MRI revealed a mass of high intensity, but no intraductal spread was detected (coronal section).

A 41-year-old Japanese woman who had a mass in her left breast was admitted to our hospital for further assessment. Physical examination revealed an irregular mass in the lateral upper quadrant of the left breast, approximately 2.5 cm in diameter. No dimpling or palpable axillary and supraclavicular lymph nodes were detected. Results of laboratory tests were all within the reference range. Mammography revealed a focal asymmetric density in the lateral upper quadrant of the left breast (Figure 1), and ultrasonography showed a heterogeneous hypoechoic mass with an ill-defined margin, measuring 3.4 × 1.1 × 3.0 cm (Figure 2). Enhanced magnetic resonance imaging (MRI) revealed a mass of high intensity, but no intraductal spread was detected (Figure 3). Further examinations, including computed tomography of the thorax and abdomen and bone scintigraphy, showed no signs of metastatic lesions. Fine-needle aspiration cytology revealed malignancy, suggesting invasive ductal carcinoma.

**Figure 4:**
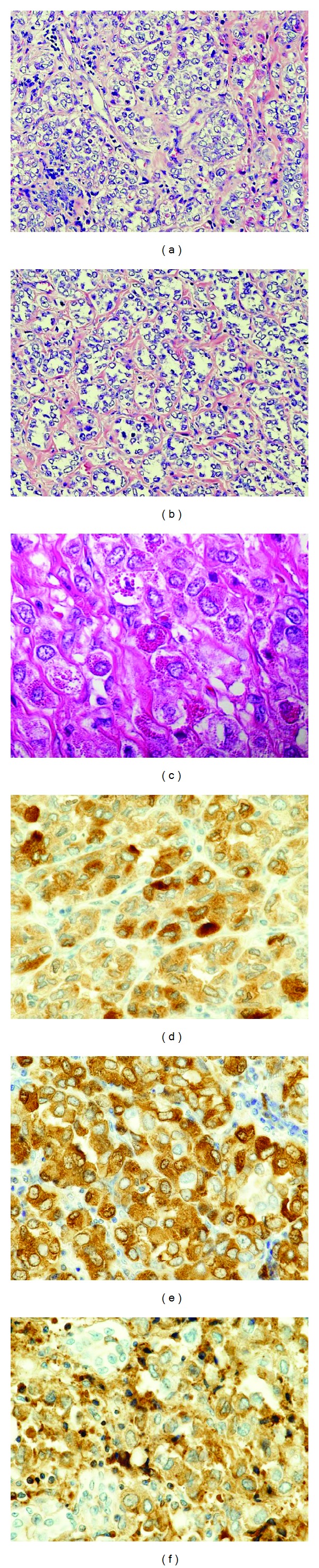
Microscopic findings showed that the tumor had an infiltrative growth pattern with a combination of solid (a), trabecular, and microglandular (b) features. Many of the tumor cells had clear cytoplasm containing large, coarse, and bright red granules which resemble acinar cells of the salivary glands (c). Immunohistochemical staining showed that most of the tumor cells stained strongly for amylase (d), lysozyme (e), and *α*1ACT (f).

The case was diagnosed as T2N0M0, stage IIA breast cancer, and lumpectomy and sentinel lymph node biopsy were therefore performed. Grossly, the specimen revealed a 3.5 × 3.0 × 2.0 cm, white-yellow-colored tumor with an ill-defined border and a rubbery consistency. Histologically, the tumor cells had round-to-oval, displaced nuclei with a striking single nucleolus and clear cytoplasm, many of which contained large, coarse, and bright red granules resembling zymogen granules of the acinar cells of the salivary gland. They showed an infiltrating growth pattern with a combination of solid, trabecular, and microglandular features (Figure 4). The nuclear grade of the tumor cells was determined to be grade 2. Lymphatic permeation was occasionally seen, but the sentinel lymph node was free of metastasis. 

Immunohistochemically, most of the tumor cells stained strongly for amylase, lysozyme, *α*-1-antichymotrypsin (*α*1ACT) (Figure 4), epithelial membrane antigen (EMA), and S-100 protein and also showed positive for cytokeratin 7 and E-cadherin. Estrogen and progesterone receptors and human epidermal growth factor receptor 2 (HER2) protein were triple negative. From the above results we could interpret the tumor as acinic cell carcinoma of the breast.

Because the patient was diagnosed as having invasive breast cancer with a triple-negative phenotype, postoperative radiotherapy (50 Gy-/25 fractions) followed by adjuvant chemotherapy (TC: docetaxel 75 mg/m^2^ and cyclophosphamide 600 mg/m^2^ administered intravenously every 3 weeks for 4 cycles) was administered. Although the follow-up period to date has been short (3 years), there have thus far been no signs of recurrence. 

## 3. Discussion

No more than 15 cases of ACC of the breast (including this case) have been reported since it was first described in 1996 as a rare variant of breast carcinoma showing morphological features resembling those of salivary glands [1]. These cases are summarized in Table 1. ACC of the breast affects women between 20 and 80 years of age (mean, 54.2 years; with a single case involving a male patient). It generally presents as a palpable nodule ranging from 2 to 6 cm in size although 1 case involved a nonpalpable mass that was only discovered by mammography [3]. Several studies have discussed the usefulness of diagnostic imagings for ACC, although their findings have sometimes differed [1, 2, 8–10]. For example, mammography showed a well-defined mass in some cases [1, 8, 10] but no abnormal findings in another [9]. Ultrasonography revealed an intracystic tumor in only male patient yet described [2]. In our case, mammography revealed focal asymmetric density, and ultrasonographic findings resembled those of ductal carcinoma in situ. Thus, at present, it seems that there are no specific imaging findings that characterize this tumor type. Findings regarding tumor spread are similarly inconsistent. Lymph node metastasis was observed in 4 cases, and 3 cases showed nodal involvement upon recurrence with additional local, liver, and lung metastases. Only 1 patient died of the tumor, suggesting a relatively favorable prognosis for this tumor type although followup was limited to a maximum of 10 years [7]. 

As the breasts and the salivary glands are known to share many similarities with respect to embryology [11], ACC of the breast is similar to its salivary gland counterpart at the morphological, immunohistochemical, and ultrastructural levels. The tumor in this case was an infiltrating solid, trabecular, and microglandular pattern and, in most ACC, the tumor cells were immunohistochemically positive for amylase, lysozyme, *α*1ACT, S-100 protein, and EMA but were negative for estrogen receptor, progesterone receptor, and HER2 protein (Table 2). 

In general, breast carcinoma lacking HER2 and the estrogen and progesterone receptors (triple-negative breast cancer TNBC) is more aggressive than other disease subtypes [12, 13]. In contrast, ACC of the salivary glands is said to be a low-grade malignant neoplasm [14–16]. Therefore, it seems that ACC of the breast has characteristics similar to those of salivary gland, even if it is of the TNBC subtype. Although 1 patient was previously reported to have died as a result of this tumor type, standard adjuvant chemotherapy for breast cancer might not be always necessary. Several studies have reported that sporadic TNBC shares clinical and pathological features with hereditary *BRCA1*-related breast cancers [17–20], and, more recently, a case of a *BRCA1* mutation carrier with an ACC of the breast was reported [21]. Therefore, further studies are necessary to determine the optimal therapeutic strategy for these tumors.

In summary, ACC of the breast is a rare variant of breast carcinoma that has been suggested to have a good prognosis even though it is often of the TNBC subtype. Currently, there are no characteristic diagnostic imaging findings for this disease, and immunohistochemical examination is important in making an accurate diagnosis. Further studies are needed to elucidate the biological characteristics of ACC of the breast. 

## Figures and Tables

**Table 1:** Clinical characteristics of acinic cell carcinoma of the breast.

Nr.	References	Age (years)	Gender	Tumor size (mm)	LN metastases	Operation	Adjuvant therapy	Follow-up (years)	Recurrence
1	Roncaroli et al. [1]	42	F	30	1/18	Bt + Ax	CT	5	—
2	Shimao et al. [2]	23	M	48	—	Bt + Ax	Not known	2.8	—
3	Damiani et al. [3]	35	F	40	2/20	Bt + Ax	CT	1	—
4	[3]	63	F	50	Not known	BCS	—	4	Local
5	[3]	55	F	20	Not known	BCS	—	Not known	Not known
6	[3]	64	F	33	0/8	BCS + Ax	—	1	—
7	[3]	80	F	20	Not known	BCS	HT	1	—
8	Schmitt et al. [4]	79	F	45	0/23	Bt + Ax	RT	1.7	—
9	Coyne and Dervan [5]	49	F	20	2/11	Bt + Ax	CT	3	Liver (died)
10	Elster et al. [6]	48	F	30	0/6	BCS + Ax	CT + RT	Not known	Not known
11	Peintinger et al. [7]	36	F	35	0/15	BCS + Ax	CT + RT	10	Lung
12	Tanahashi et al. [8]	80	F	30	—	Bt + SN	Not known	1.8	—
13	Chang et al. [9]	39	F	55	1/Not known	BCS + Ax	Not known	Not known	Not known
14	Choh et al. [10]	79	F	27	—	BCS + SN	RT	0.8	—
15	Present case	41	F	25	0/1	BCS + SN	CT + RT	3	—

Bt:  total mastectomy; BCS: breast-conserving surgery; Ax: axillary lymph nodes dissection; SN: sentinel lymph node biopsy; CT: chemotherapy; HT: hormone therapy; RT: radiotherapy.

**Table 2:** Immunohistochemical characteristics of acinic cell carcinoma of the breast.

Nr.	References	Amylase	Lysozyme	*α*1-ACT	S-100	EMA	ER	PgR	HER2
1	Roncaroli et al. [1]	ND	+	+	+	+	−	−	ND
2	Shimao et al. [2]	+	ND	ND	+	+	+	ND	ND
3	Damiani et al. [3]	+	+	+	+	+	−	−	ND
4	[3]	+	+	+	+	+	−	−	ND
5	[3]	+	+	+	+	+	−	−	ND
6	[3]	+	+	+	+	+	−	−	ND
7	[3]	+	+	+	+	+	−	−	ND
8	Schmitt et al. [4]	ND	+	ND	−	+	−	−	−
9	Coyne and Dervan [5]	ND	+	+	+	ND	ND	ND	ND
10	Elster et al. [6]	ND	+	ND	+	+	−	−	−
11	Peintinger et al. [7]	ND	+	+	+	+	−	−	ND
12	Tanahashi et al. [8]	+	−	ND	−	ND	−	−	−
13	Chang et al. [9]	ND	+	+	+	+	−	−	ND
14	Choh et al. [10]	ND	ND	ND	ND	ND	ND	ND	ND
15	Present case	+	+	+	+	+	−	−	−

α1-ACT: alpha-1-antichymotrypsin; EMA: epithelial membrane antigen; ER: estrogen receptor; PgR:  progesterone receptor; HER2: human epidermal growth factor receptor 2; ND: not done.
